# MicroRNA Expression Profile in the Prenatal Amniotic Fluid Samples of Pregnant Women with Down Syndrome

**DOI:** 10.4274/balkanmedj.2017.0511

**Published:** 2018-03-15

**Authors:** Emin Karaca, Ayça Aykut, Biray Ertürk, Burak Durmaz, Ahmet Güler, Barış Büke, Ahmet Özgür Yeniel, Ahmet Mete Ergenoğlu, Ferda Özkınay, Mehmet Özeren, Mert Kazandı, Fuat Akercan, Sermet Sağol, Cumhur Gündüz, Özgür Çoğulu

**Affiliations:** 1 Department of Medical Genetics, Ege University School of Medicine, İzmir, Turkey; 2 Department of Obstetrics and Gynecology, Ege University School of Medicine, İzmir, Turkey; 3 Clinic of Obstetrics and Gynecology, İzmir Ege Maternity and Women’s Diseases Training Research Hospital, İzmir, Turkey; 4 Department of Medical Biology, Ege University School of Medicine, İzmir, Turkey

**Keywords:** Down syndrome, microRNAs, amniotic fluid

## Abstract

**Background:**

Down syndrome, which is the most common human chromosomal anomaly that can affect people of any race and age, can be diagnosed prenatally in most cases. Prenatal diagnosis via culture method is time-consuming; thus, genetic analysis has thus been introduced and is continually being developed for rapid prenatal diagnosis. For this reason, the effective use of microRNA profiling for the rapid analysis of prenatal amniotic fluid samples for the diagnosis of Down syndrome was investigated.

**Aims:**

To evaluate the expression levels of 14 microRNAs encoded by chromosome 21 in amniotic fluid samples and their utility for prenatal diagnosis of Down syndrome.

**Study Design:**

Case-control study.

**Methods:**

We performed invasive prenatal testing for 56 pregnant women; 23 carried fetuses with Down syndrome, and 33 carried fetuses with a normal karyotype. Advanced maternal age and increased risk for Down syndrome in the screening tests were indications for invasive prenatal testing. The age of gestation in the study and control groups ranged between 17 and 18 weeks. The expression levels of microRNA were measured by real-time polymerase chain reaction.

**Results:**

The expression levels of *microRNA-125b-2*, *microRNA-155*, and *microRNA-3156* were significantly higher in the study group than in the control group.

**Conclusion:**

The presence of significantly dysregulated microRNAs may be associated with either the phenotype or the result of abnormal development. Further large-scale comparative studies conducted in a variety of conditions may bring novel insights in the field of abnormal prenatal conditions.

Down syndrome (DS) is the most common aneuploidy that is compatible with life and has been reported to affect approximately 1 in 700 births in the general population (1). The most common risk factor of DS is advanced maternal age (AMA). The clinical presentation of DS varies according to the associated phenotype. The presence of characteristic facial dysmorphology, small and hypocellular brain, and intellectual disability can be observed to some extent in all individuals with DS. Trisomy 21 is also a risk factor for a number of diseases, such as congenital heart disease, which is the most common disease that occurs in 40%-50% of patients with DS (2). Leukemia, autoimmune diseases, and Hirschsprung disease are the other common clinical presentations of DS. More than 80 clinical features occur more frequently in individuals with DS than in the general population (3). These complicated and varied phenotypes are generally thought to result from the imbalance between the expression of trisomic genes on chromosome 21 and disomic genes on other chromosomes, resulting into abnormal gene dose. For this reason, studies on DS have mainly focused on the changes in the expression of genes on chromosome 21 or in the noncoding molecules, such as microRNA (miRNA), in the tissues of individuals with DS. miRNAs are small, noncoding RNA molecules that comprise 18-22 nucleotides and have critical functions throughout the epigenetic regulation of the genome. Recent evidence has shown that miRNAs play important roles in various physiological and pathological processes, such as cell differentiation, cell proliferation, development, apoptosis, and oncogenesis (1). Reproductive genetic counseling on the prenatal diagnosis of DS involves conventional diagnostic methods, including chorionic villus biopsy, amniotic fluid sampling, and fetal blood sampling. In addition, the contribution of miRNAs encoded by extra chromosomal material in relatively common human aneuploidy syndromes, such as trisomy 21, to the pathogenesis of their related phenotypes has been previously described (4,5). This study aimed to compare pregnancies with and without DS in terms of the expression levels of 14 miRNAs encoded by chromosome 21 in 56 prenatal samples.

## MATERIALS AND METHODS

### Patients

The study included 56 pregnant women aged 27-43 years who were followed-up at 17-18 weeks of gestation; 23 women carried fetuses with DS, and 33 women carried healthy fetuses (with a normal karyotype). Two groups of prenatal samples were analyzed: group 1 comprised prenatal samples from pregnant women carrying fetuses with DS, and group 2 comprised prenatal samples from pregnant women carrying fetuses without DS. Pregnant women with any history of systemic disease or infection or those in group 1 who carried fetuses with conditions not compatible with DS were excluded from the study. Trisomy 21 was confirmed by karyotyping 20 well-spread metaphases following amniotic fluid sampling. No mosaic case was included in the study. This study was approved by the ethical committee, and written informed consent was obtained from each subject.

### Amniotic fluid culture and miRNA expression analysis

Amniotic fluid sampling was used for the study. Cell cultures were prepared using three flasks; the first two flasks were separated for routine analysis, and the third flask was used for the study. Quantitative fluorescence polymerase chain reaction (PCR) was conducted to exclude maternal contamination in the samples. A total of 5 mL of cell suspension was used for real-time (RT)-PCR (Roche Light Cycler, Mannheim, Germany) to detect and quantify the expression of mature miRNA in the samples. Frozen samples were dissolved and homogenized. The miRNeasy RNA isolation kit (Qiagen, Hilden, Germany) was used for isolation and enrichment of the miRNAs, according to the manufacturer’s instructions. A total of 10 mg of RNA was tested using PureLink^®^ RNA Mini Kit, according to the manufacturer’s guidelines. The TaqMan^®^ probe, which contained a 5’ reporter dye and a 3’ nonfluorescent quencher, was used for RT-PCR. TaqMan^®^ microRNA assay was performed for quantification using two-step RT-PCR. Specific miRNA primer complementary DNA samples were obtained from total RNA samples that were subjected to reverse transcription using the TaqMan^®^ miRNA Reverse Transcription Kit. In the second step, PCR products were amplified using the TaqMan^®^ miRNA Assay and the TaqMan^®^ Universal PCR Master Mix.

The expressions of 14 miRNAs [i.e., human chromosome (hsa)-let-7c, hsa-mir-125b-2, hsa-mir-155, hsa-mir-3118, hsa-mir-3156, hsa-mir-3197, hsa-mir-3648, hsa-mir-3687, hsa-mir-4327, hsa-mir-4759, hsa-mir-4760-3p, hsa-mir-548x, hsa-mir-802, and hsa-mir-99a] encoded by chromosome 21 were evaluated; u6-snRNA was used as the control sample for normalization.

### Statistical analysis

Student’s t-test, regression analysis, and fold change comparisons were performed. Fold changes were calculated using RT2 Data Analysis (http://pcrdataanalysis.sabiosciences.com/pcr/arrayanalysis.php) and described as the ratio of the average values in the control and study samples. A two-fold change with a p value of <0.05 was considered statistically significant. The average values and standard deviations of the current transformer (CT) and ΔCT (CT of target - CT of reference) were calculated. Moreover, the ΔΔCT value (ΔCT of the test sample - ΔCT of the calibrator sample) was calculated. Student’s t-test was used to compare the average values between two groups. The fold change was calculated to validate the p values obtained from Student’s t-test using ΔΔCT values.

## RESULTS

**Figure 1:**
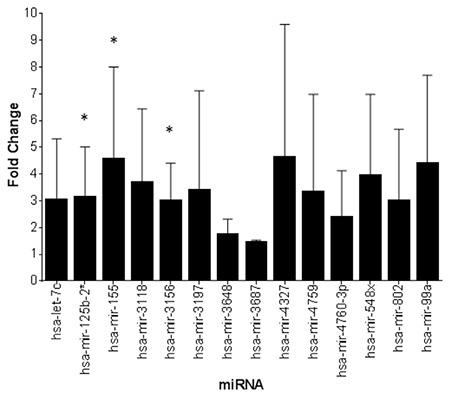
Differential expression of selected miRNAs in cell cultures from the Down syndrome and control samples. *Significantly increased expressions; miRNA: microRNA; hsa: human chromosome

This study included 56 pregnant women. The study and control groups had a mean age of 37.00±4.59 years and 37.00±3.99 years, respectively, and a median age of 36.38 and 36.40 years, respectively. The indications for prenatal testing included AMA in 73% (n=16), high risk in maternal plasma screening (HRMPS) in 18.9% (n=5), and abnormal finding in the sonographic examination in 10.8% (n=2) of all subjects. In the control group, AMA and HRMPS were indications for prenatal testing in 75% and 25% of subjects, respectively. Three of 14 miRNAs (miR-125b-2, miR-155, and miR-3156) were found to be overexpressed in the study group compared with those in the control group. Table 1 and Figure 1 show the p values and the fold changes of all miRNAs.

## DISCUSSION

The miRNAs are a new class of nucleic acids that show promise as useful clinical biomarkers for several diseases (6,7,8). Our data revealed that the expressions of miR-125b-2, miR-155, and miR-3156 in the amniotic fluid culture were significantly higher in pregnant women carrying fetuses with DS than in the control group. The study sample size was small; however, to the best of our knowledge, our study is the first to demonstrate significant differences in miRNA expression in cell cultures between fetuses with and without DS. Many studies have evaluated the association of miRNA expression levels with a variety of pathologic conditions, including preeclampsia, fetal congenital cardiac defects, neural tube defects, fetal growth retardation, and DS (9,10,11,12,13,14,15). The miRNAs encoded by hsa 21 (i.e., hsa-miR-99a, let-7c, miR-125b-2, miR-155, and miR-802) have been proven to correlate with the complex and variable phenotypes of DS and were shown to be overexpressed in the heart, frontal cortex, and hippocampus of fetuses with DS as found in our study (4,16,17,18,19). Kuhn et al. (17) analyzed miRNA expression profiles of DS hippocampus specimens and verified that miRNAs encoded by chromosome 21 were overexpressed in the DS brain. Szulwach et al. (19) showed the overexpression of miR-155 in the fibroblasts of discordant monozygotic twins and also confirmed that miR-155 was associated with cognitive impairment in individuals with DS by regulation of the *MeCP2* gene (18,20). In addition, the target genes of miR-155 were reported to be involved in hematopoietic and myeloproliferative disorders (21); in particular, hsa-miR-155 was found to be important in the development of immunocytes during inflammation through regulation of the hsa-miR-155 target genes. hsa-miR-155 is necessary for the normal immune function of B lymphocytes, T lymphocytes, and dendritic cells (22). The deficiency of hsa-miR-155 makes CD4+ T cells more prone to T helper (Th)-2 differentiation than Th-1 differentiation by forming high-affinity immunoglobulin G antibodies, which decreases the number of germinal center B cells produced (23). However, higher levels of this miRNA may be associated with abnormal conditions, such as immunologic and cardiovascular problems or abnormal cell proliferation, in children with DS (24,25,26). In this study, miR-125, which is a highly conserved miRNA in several species, was found to be overexpressed in the DS samples. In humans, hsa-miR-125b-1, hsa-miR-125b-2, and hsa-miR-125a are the three homologs of miR-125. The proteins that play an important role in apoptosis, hematopoietic differentiation, and innate immunity have been shown as the targets of miR-125b. It can also be highly expressed in specific subtypes of myeloid and lymphoid leukemia and is advantageous in providing resistance to apoptosis. It is accepted as an oncomiR in the hematopoietic system (27). In contrast, miR-125b-2 plays an important role as a tumor suppressor and has been shown to reduce the incidence of solid tumors in individuals with DS carrying hsa-miR-99a and let-7c (28). Approximately 1%-2% of children with DS develop acute megakaryoblastic leukemia, mostly before the age of 5 years. In the present study, we found higher expression levels of miR-125b in the amniotic fluid culture from the study group than from the control group. This finding may indicate the role of miRNAs in the development of cancer in the early period of life. Dong et al. (29) also reported the presence of miR-125b-2 in cases with recurrent spontaneous abortions. Therefore, this finding can also be used for high-risk pregnancies. To the best of our knowledge, this is the first study to reveal the association between miR-3156 and DS prenatally, as there is no information in literature to date. The effect of miR-3156-3p on the carcinogenetic processes of CC cell lines has been shown in a recent study (30). However, the function of miR-3156-3p remains unknown, and there are insufficient studies on miR-3156-3p. One limitation of this study was the relatively small sample size. Nevertheless, our findings may provide valuable insights regarding the use of miRNAs in the prenatal diagnosis of DS. In conclusion, the results should be considered cautiously for prenatal diagnosis of DS because the method is time-consuming and invasive. However, this study can contribute to the literature mainly for the following three reasons:

- The possible associations of our results with abnormal findings in fetuses with DS, as discussed. Notably, the mechanism of regulation of miRNAs in the development of DS cultured cells and the association between miRNAs encoded by chromosome 21 and the various phenotypes of DS should be further investigated;

- The high expression levels of miRNAs in fetal cell cultures, particularly associated with the genetic disease of interest, may open new horizons in the investigation of pathological conditions; and

- The miRNAs in prenatal samples show promise as novel diagnostic biomarkers, not only for DS but also for other genetic diseases.

Further studies evaluating miRNA in fetal cell cultures may provide important inputs for the detection of other fetal pathologies. Expression profiling is likely to increase our understanding of miRNAs and their roles in the development of fetuses with DS.

## Figures and Tables

**Table 1:**
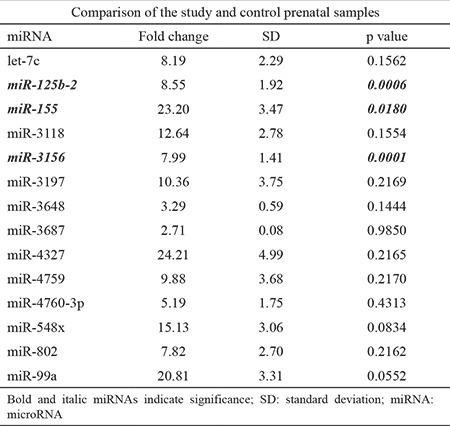
Fold change and p values of the miRNA expressions in the prenatal samples
